# Pharmacokinetic (PK) and pharmacodynamic analyses of once- and twice-daily darunavir/ritonavir (DRV/r) in the ODIN trial

**DOI:** 10.1186/1758-2652-13-S4-P185

**Published:** 2010-11-08

**Authors:** V Sekar, G De La Rosa, T Van de Casteele, S Spinosa-Guzman, P Vis, RMW Hoetelmans

**Affiliations:** 1 Tibotec Inc, Titusville, NJ, USA; 2 Tibotec BVBA, Beerse, Belgium

## Background

In the Phase III, randomised, open-label ODIN trial, treatment-experienced HIV-1-infected adults with no screening DRV resistance-associated mutations received DRV/r 800/100mg qd or DRV/r 600/100mg bid (both arms + ≥2 NRTIs). At Week 48, 72.1% qd vs 70.9% bid patients achieved HIV-1 RNA <50 copies/mL (95% CI = -6.1 to 8.5%, p<0.001; ITT-TLOVR), confirming non-inferiority of DRV/r qd. The relationship between DRV PK and efficacy and safety following treatment with DRV/r is explored.

## Methods

Sparse blood sampling for PK evaluations was taken at Weeks 4, 8, 24 and 48 to determine DRV trough concentrations (C_0h_) and exposure (AUC_24h_, calculated as AUC_12h_ x 2 for bid) using a population pharmacokinetic model. Relationships between PK parameters and efficacy (change in log_10_ HIV-1 RNA and virological response [HIV-1 RNA <50 copies/mL]) were assessed using ANCOVAs. Relationships between PK parameters and occurrence of adverse events of interest and laboratory lipid abnormalities were evaluated using descriptive statistics.

## Results

PK data were available for 280 DRV/r qd patients and 278 bid patients. Median (range) C_0h_ was 1896 (184-7881) ng/mL for DRV/r qd and 3197 (250-11,865) ng/mL for DRV/r bid. Median (range) AUC_24h_ for DRV/r qd was 87,788 (45,456-236,920) ng.h/mL and 109,401 (48,934–323,820) ng.h/mL for bid. No relevant relationships were observed between DRV PK and efficacy: changes from baseline in HIV-1 RNA log_10_ copies/mL at Week 48 for pooled data by DRV AUC_24h_ quartile ranges (≤79,576; 79,577-100,376; 100,377-119,356; >119,356 ng.h/mL) were -2.06, -2.22, -2.19, and -2.08 log_10_ copies/mL, respectively. The % patients achieving HIV-1 RNA <50 copies/mL by these quartile ranges were 82.0%, 88.5%, 82.6% and 76.5% (observed data), respectively. In a logistic regression analysis adjusting for baseline viral load, AAG levels and number of sensitive NRTIs in the optimised background regimen, there were no relevant relationships between PK and virological response. No apparent relationships were observed between DRV PK and occurrence of rash-, cardiac-, GI-, liver-, lipid- and glucose-related AEs or laboratory lipid abnormalities.

## Conclusions

Dosing with DRV/r 800/100mg qd resulted in lower C_0h_ and AUC_24h_ compared to DRV/r 600/100mg bid; however, comparable efficacy between DRV/r qd and bid confirmed adequate DRV concentrations were achieved following qd dosing. No relevant relationships were observed between DRV PK and efficacy or safety at Week 48.

